# Editorial: What can simple brains teach us about how vision works

**DOI:** 10.3389/fncir.2015.00051

**Published:** 2015-09-29

**Authors:** Davide Zoccolan, David D. Cox, Andrea Benucci

**Affiliations:** ^1^Visual Neuroscience Lab, International School for Advanced StudiesTrieste, Italy; ^2^Department of Molecular and Cellular Biology and Center for Brain Science, Harvard UniversityCambridge, MA, USA; ^3^Laboratory for Neural Circuit and Behavior, RIKEN Brain Science InstituteWako City, Japan

**Keywords:** rodent, development, motion processing, object recognition, illusory contours

*Vision* is the process of extracting behaviorally-relevant information from patterns of light that fall on retina as the eyes sample the outside world. Traditionally, non-human primates have been viewed by many as the animal model-of-choice for investigating the neuronal substrates of visual processing, not only because their visual systems closely mirror our own (e.g., Orban, 2008; Nassi and Callaway, 2009 for a review), but also because it is often assumed that “simpler” brains lack advanced visual processing machinery. However, this narrow view of visual neuroscience ignores the fact that vision is widely distributed throughout the animal kingdom, enabling a wide repertoire of complex behaviors in species from insects to birds, fish, and mammals.

Recent years have seen a resurgence of interest in alternative animal models for vision research, such as rodents (see Huberman and Niell, 2011; Zoccolan, 2015 for a review). This resurgence is partly due to the availability of increasingly powerful experimental approaches (e.g., optogenetics and two-photon imaging) that are challenging to apply to their full potential in primates. Meanwhile, even more phylogenetically distant species such as birds, fish, and insects have long been workhorse animal models for gaining insight into the core computations underlying visual processing (see Baier, 2000; Bilotta and Saszik, 2001; Borst et al., 2010; Aptekar and Frye, 2013 for a review). In many cases, these animal models are valuable *precisely because* their visual systems are simpler than the primate visual system. Simpler systems are often easier to understand, and studying a diversity of neuronal systems that achieve similar functions can focus attention on those computational principles that are universal and essential.

This Research Topic provides a survey of the state of the art in the use of non-primate models of visual functions. It includes original research, methods articles, reviews, and opinions that exploit a variety of animal models (including rodents, birds, fishes and insects) to investigate visual function. The experimental approaches covered by these studies range from psychophysics and electrophysiology to histology and genetics, testifying to the richness and depth of visual neuroscience in non-primate species. Below, we briefly summarize the contributions to this Research Topic.

## Rodent studies

Roughly half of the articles in this Research Topic (6 research studies and 4 reviews) focus on the visual system of two rodent species more commonly used as laboratory animals: rats and mice. Following a trend that has been established over the past 6–7 years, the mouse studies investigate tuning properties of visual neurons in low-level visual centers through *in-vivo* electrophysiology and, in one case, genetic manipulation (LeDue et al., 2013; Liu et al., 2014), while the rat studies explore higher-level perceptual functions (such as pattern discrimination) through visual psychophysics and, in one case, *in-vivo* neurophysiology (Meier and Reinagel, 2013; Reinagel, 2013; Rosselli et al., 2015; Vermaercke et al., 2015). The reviews focus on the role of rats and mice as models of development and plasticity of the visual system (Bonaccorsi et al., 2014; Priebe and McGee, 2014), and on the comparison among the visual cortical organizations of rodents, primates and other species (Homman-Ludiye and Bourne, 2014; Laramée and Boire, 2015).

LeDue et al. (2013) investigate the stimulus-dependence properties of contrast adaptation in mouse primary visual cortex (V1). When a high-contrast stimulus is shown even for a few seconds, the response amplitude of V1 primate neurons to subsequent stimuli is weakened. LeDue et al., report the same stimulus-specificity in mouse V1. This observation opens the possibility that network, synaptic, and intrinsic cellular mechanisms contributing to contrast adaptation operate in mouse V1 in a similar way as in higher mammals.

Liu et al. (2014) present a paper on mouse superior colliculus (SC) and take full advantage of transgenic technologies. In particular, the authors study the receptive fields (RFs) of SC neurons. Such RFs are shaped by converging retinal on- and off-pathways, guided by molecular guidance cues (e.g., EphAs and ephrin-As). In addition to these cues, retinal function also plays a critical role. Knockout mice where retinal activity is altered during development (nAChR-β2−∕−) have SC neurons with severely disrupted direction and orientation selectivity. Liu et al., show that knocking out guidance cues (ephrin-A knockout) has very little impact on the RFs, making them just slightly larger—an elegant example of how transgenic technologies can help dissect the relative contribution of activity-dependent mechanisms and genetic programs.

In rats, Meier and Reinagel (2013) investigate whether the detection of a centrally-presented grating is similarly affected in rats and humans by the concomitant presentation of two flanking gratings. They report that, in both species, the flankers with the greatest impact on target detection are those that are collinear to the target (i.e., they are located and oriented to sit along a virtual line passing through the three stimuli). However, while collinear flankers maximally impair detection in rats, they maximally improve it in humans. This implies that rats, like humans, are sensitive to higher-order configurations of oriented elements, but the sign of this phenomenon is the opposite in the two species. This raises intriguing questions about differences between neuronal mechanisms that, in rodents and primates, underlie spatial integration of visual features, spatial attention and center-surround stimulus interactions.

In a second study, Reinagel (2013) investigates whether visual sensory decisions in rats are constrained by the speed-accuracy trade-off that is typical of primate vision. The author reports that rat accuracy in discriminating static images increases with reaction time. Additionally, accuracy and speed are both modulated by task difficulty and the penalty associated with an incorrect response. This represents an interesting basis for comparing the dynamics of perceptual decisions in rodents and primates, and provides useful insights for effectively training rats in visual discrimination tasks.

Rosselli et al. (2015) also investigate the impact of stimulus discriminability on rat pattern vision, but focus on the difference between the perceptual strategies underlying the recognition of structurally similar vs. dissimilar objects across view changes (i.e., variations in position, size and orientation). They report that the pattern of diagnostic features underlying the discrimination of highly similar objects are more scattered, more view-dependent, and more subject dependent, as compared to those found in a previous study using more dissimilar disciminanda (Alemi-Neissi et al., 2013). These findings suggest that in rats, as in primates, transformation-tolerant recognition can flexibly rely on either view-invariant representations of distinctive object features or view-specific representations that are acquired through exposure to multiple object views.

Rat pattern vision is also the topic of the study of Vermaercke et al. (2015), who compare the discriminability of different pairs of visual shapes at a behavioral level with their discriminability at the neuronal level. The authors report that neuronal discriminability correlates well with behavioral discriminability only in the extrastriate visual cortical areas that are lateral to primary visual cortex (V1), but not in V1 itself (where, instead, they find a good correlation with shape discriminability at the pixel level). This suggests that rat lateral visual cortex represents behaviorally relevant shape features, in a way that could be homologous to the primate ventral stream.

Two reviews focus on the plasticity of the rodent visual system during development (Priebe and McGee, 2014) and in adulthood (Bonaccorsi et al., 2014). Priebe and McGee (2014) comment on some of the major distinctive features of the mouse early visual system: from the retina to the primary visual cortex. They then delve into the most studied form of experience-dependent plasticity in the visual cortex: ocular-dominance (OD) plasticity. Activity-dependent changes in OD patterns during the critical period have been observed in all mammals and mice are no exception. This review highlights the key genetic mechanisms involved, with special attention to the role of inhibition during the narrow critical period (P20-32) of plasticity.

Bonaccorsi et al. (2014) provide a comprehensive overview of amblyopia, with a focus on the role of perceptual learning as a possible treatment for this condition in both humans and animals. The authors discuss recent experiments in which adult amblyopic rats showed a full recovery of visual functions as a result of extensive training in a spatial frequency discrimination task. The associated decrease of the inhibition-excitation balance highlights the fundamental role that the reduction of GABAergic inhibition can play in restoring cortical plasticity and enhancing recovery of function in the adulthood. This confirms the effectiveness of rodent models in the study of visual cortical plasticity and their role in the development of new therapeutic approaches.

Two other reviews compare the anatomy, connectivity, parcellation and hierarchical organization of the visual systems of different species, with a special focus on primates and rodents (Homman-Ludiye and Bourne, 2014; Laramée and Boire, 2015). Homman-Ludiye and Bourne (2014) provide a comparative review of the studies concerning the cellular, molecular and genetic mechanisms responsible for visual cortical arealisation in a variety of mammalian species. The authors draw evidence from methodological approaches ranging from the application of anterograde and retrograde tracers, histological mapping of activity-dependent cellular markers (e.g., immediate-early genes), determination of the regulatory events that roughly define area borders during development (e.g., the graded expression of transcription factors along brain axes), and understanding of the molecular guidance cues that refine these borders into the sharp boundaries of the mature visual cortex. Overall, the review makes the point that the analysis of multiple species is important to understand the evolution and development of the mammalian visual system, with an emphasis on the experimental advantages that genetically modified mice afford.

In a similar spirit, Laramée and Boire (2015) focus on the order Rodentia, which represents over 40% of all mammalian species. This order is incredibly diverse: more than 2000 species with 1000-fold change in body size and 200-fold change in brain size. Such diversity within the same order represents a great opportunity to identify general principles of anatomical and functional organization. Laramée and Boire (2015) look at what is preserved and what is lost across species and discuss such observations in the context of theories of optimality (wiring economy, small-world networks, etc.), which is a convenient theoretical framework to reveal the underlying organizational principles.

## “Simpler” primates studies

While this Research Topic was mostly focused on non-primate systems, we included one exception: a review of what is known about the visual system of a new world monkey, the marmoset (Solomon and Rosa, 2014). In their review, the authors compare the “simpler” brain of the marmoset to that of the macaque monkey, which is still considered the benchmark model for primate vision. In this thorough and comprehensive review of the marmoset visual system, Solomon and Rosa (2014) start from the retina and end in frontal association areas, touching on sub-cortical structures as well. In this voyage through the marmoset brain, the authors discuss distinctive functional and anatomical features that make it a promising alternative to the larger, more complex macaque brain.

## Bird studies

Object recognition is a topic that is also addressed by two bird studies, one research article (Wood and Wood, 2015) and one review (Soto and Wasserman, 2014). Wood and Wood (2015) follow up on previous work exploring the visual object recognition abilities of newborn chickens (Wood, 2013). The authors rely on the innate imprinting behaviors of this species, in which the chick approaches stimuli that it has previous seen in its early life. The study reports that the animals are capable of generalizing from extremely limited exposure to visual objects—in some cases just a handful of views. These results suggest that the chicken's visual system is able to learn robust visual representations of objects from extremely little training data.

Taking a broader view on avian vision, Soto and Wasserman (2014) review the large body of work focused on the object recognition abilities of pigeons. Pigeons have long been known to exhibit sophisticated visual recognition abilities. The authors argue that many core components of object recognition behavior are found across a wide range of vertebrate species, and that birds represent a fruitful model system for studying these abilities.

## Fish and amphibian studies

High-level visual functions, such as shape processing and object recognition, are also addressed by several behavioral studies on fish in this Research Topic.

The perception of illusory shapes and boundaries is the subject of two reviews/opinions (Agrillo et al., 2013; Rosa Salva et al., 2014) and one research article (Fuss et al., 2014). Based on the observation that different groups of teleost fish exhibit both modal and amodal completion (e.g., perception of illusory contours, as in the Kanizsa figures), Agrillo et al. (2013) argue that fish represent an excellent experimental model for studying the development of gestalt principles of visual perception in newborn animals. In particular, the authors stress the potential of investigating such principles in the zebrafish, one of the main model organisms for the study of neurodevelopmental genetics.

Along these lines, Fuss et al. (2014) present new results suggesting that bamboo sharks perceive at least some illusory contour stimuli in a manner similar to how they are perceived in other non-fish species. Bamboo sharks generalize training with visual shapes to their equivalent Kanizsa figures, though results with some illusions, such as Mueller-Lyer figures, are less clear. These results speak both to the universality of certain mechanisms of contour perception and to the ability to probe detailed behavior in a wide range of fish species.

In their review, Rosa Salva et al. (2014) stress the important advantages of working with fish for comparative studies of brain evolution. Fish diverged from other vertebrates about 450M years ago, and diversified into a collection of taxa. This diversification makes fish an excellent model system to study how recognized homologies have evolved using diverse neural resources and substrates. The authors discuss a number of complex visual processing functions in relation to visual illusions, 2nd order motion, perceptual binding, attentional prioritization, etc. Together, these observations challenge the assumption that a complex neural circuitry (e.g. an associative cortex) is needed for adaptive object perception.

Two other behavioral studies investigate object categorization (Newport et al., 2014) and spectral sensitivity (Siebeck et al., 2014) in fish models. Newport et al. (2014) assess the ability of the archer fish to categorize objects using a range of challenging psychophysical tasks. While it is difficult to train these fish to perform some more complicated tasks, such as match-to-sample and odd-one-out tasks, the fish are able to robustly learn two-alternative forced choice tasks, providing a powerful window into the visual abilities of this species.

Siebeck et al. (2014) examine luminance perception in reef fish, focusing in particular on spectral sensitivity of luminance vision. They find that, as in many terrestrial vertebrates, long and medium wavelength cones contribute to luminance perception, but short wavelength (blue) cones do not.

Finally, one research article uses the retina of an amphibian, the bullfrog, to study the effect of dopamine on the processing of visual information (Xiao et al., 2014). Dopamine is synthesized and released by interplexiform and amacrine cells in the bullfrog retina and is known to exert a number of important modulatory effects on retinal responses. The authors, by systematically changing the duration of visual stimuli and using an information-theoretical approach, dissect the complex role of dopamine in the encoding of stimulus duration.

## Insect studies

The Topic also includes two articles focusing on motion perception and visual tracking behavior in insect models (Aptekar et al., 2014; Egelhaaf et al., 2014). Aptekar et al. (2014) present methods and software for probing the motion processing system of Drosophila. This work represents just one example of the high degree of sophistication in stimulus generation and data analysis that exists for interrogating the visual system in insets.

Finally, in their review, Egelhaaf et al. (2014) take a broader perspective on motion processing in insects, noting that their motion detection system is sensitive to non-motion visual properties such as texture, and that these properties may reflect the adaptation of the visual system to its environment and the needs of the animal. The review represents an interesting perspective on how a simple visual system and the statistics of the natural environment can interact to enhance the readout of behaviorally-relevant cues, such as the location of nearby objects, while suppressing the representation of less-relevant distal cues.

## Concluding remarks

As the depth and breadth of the contributions to this Research Topic attest, “simpler” brains have a great deal to teach us about vision. From insects to fish, birds, amphibians, and finally mammals, research aimed at understanding vision in simple animal models is flourishing.

### Conflict of interest statement

The authors declare that the research was conducted in the absence of any commercial or financial relationships that could be construed as a potential conflict of interest.
